# Dioxydiamidoarsenobenzol

**Published:** 1911-01-07

**Authors:** 


### SPECIAL ARTICLES.

### DIOXYDIAMIDOARSENOBENZOL.

### THE PRESENT POSITION OF "606."

The remedy for syphilis, which for the last six months has been so prominently before the medical, and even before the lay public, and which upon the Continent has become a popular subject of humorous cartoon, is now for the first time placed upon the market by Meister, Lucius, and Bruning, the firm to which Ehrlich has from the beginning entrusted its manufacture. The fact that the drug, which for commercial purposes will be known by the name "Salvarsan," is to be produced by the same firm which has up to the present supplied all the material used for the preliminary clinical work is important, for it is a guarantee that the substance to be supplied commercially will be properly prepared.

### The History of "606."

It is now about nine months since the first communication was made by Professor Alt to the medical profession of the results obtained from the use of this remedy, but at the present moment many thousands of cases have already been treated. It may therefore be considered opportune to attempt to define in some measure the general attitude of the medical world towards it. The history of "606" resembles that of most new remedies, and may be divided into three periods; firstly one of enthusiasm, followed by a second of criticism and reaction, and finally by a stage in which the new drug finds its established place in therapeutics with its indications and limitations accurately defined. Many drugs, indeed, introduced amid great pomp and circumstance, never reach the final stage at all, but fade quickly into obscurity. Such a fate is not likely to overtake "606." No one who has had opportunities of using it denies that it speedily diminishes and usually removes active syphilitic lesions, and the real question at issue is whether it ought to supplant mercury as the routine treatment and whether we can expect it to stamp out the disease in the same way that by other methods we have been able to stamp out small-pox. The goal which Ehrlich has had in view during all his experiments on the organic compounds of arsenic has been to find a substance which will cure syphilis in one dose. Dioxydiamidoarsenobenzol, or to give it its commercial title "Salvarsan," apparently does so in many cases. Some cases, on the other hand, it fails to affect at all, some it affects only imperfectly, while others, again, relapse quickly after an apparent success, and it is as yet too soon to prophesy that there will be no late relapses. No doubt many of the relapses which have occurred among the early cases treated were due to the administration of too small a dose; now that we know that even as much as 22 grains may be given with safety to an adult it is probable that relapses will become less frequent. One point on which almost all observers are agreed is the speed with which this drug acts: lesions which under mercury would take a month or more to disappear vanish in less than a week under the charm of "606." Even if a permanent cure is not attained in this time the acceleration in clinical recovery is of vast importance to the community at large, because it reduces so enormously the period during which the patient remains a source of infection to others, and it is perfectly reasonable to suggest that the permanency of the cure should be consolidated by the administration of mercury, a course which has already been adopted by certain German physicians, whose results will be awaited with great interest.

### The Dangers of the New Remedy.

What, then, are its special dangers, and are there any special difficulties in its administration? Up to the present twelve deaths have been recorded as following an injection. This is not a great number considering that many thousands of cases have now been treated, some already moribund, but in a few there is no doubt that the *exitus lethalis* was accelerated by its use. Still, as our experience of the drug accumulates we shall be able to avoid the cases which are likely to be dangerous, and thus the peril of such accidents will be minimised. There seems no doubt that "Salvarsan" is far less toxic than any of the arsenic compounds which are its chemical parents—for example, atoxyl and arsacetin which have achieved a very bad reputation for causing optic atrophy. Kowalevski has, it is true, published a case of optic neuritis occurring, at an interval of some weeks, after an injection of Salvarsan, but this he regarded as a syphilitic relapse and succeeded in curing it by mercurial treatment, and several ophthalmologists record excellent results after the use of it in specific disease of the eye. We have heard of no case of nerve deafness due to it, as after the use of arsacetin.

### How to Administer "606."

Finally we turn to the difficulty of administration, and to the reaction and painful shock which may ensue. The substance is sent out in single doses in sealed and partially-exhausted glass capsules, and it has to be prepared carefully for injection. These preparations are rather awkward for those who are not accustomed to the manipulations of the chemical laboratory, and if attempted for the first time are very likely to lead to the waste of a dose or two, or even to the injection not of "Salvarsan," but of its decomposition products which may be extremely toxic. In the original method employed by Alt the injection was large in bulk, and was also strongly alkaline, so that it was extremely painful; in fact, in one case the shock was so severe that death ensued. The presence of severe nervous disease is one of the chief contra-indications to the use of "606." Respiratory failure occurs, if at all, within an hour or two of the injection. The extreme pain of the injection, which, curiously enough, men seem to feel much more than women, has been dealt with in several ways. Michaelis advocates the use of a neutral medium in which the drug is held finely suspended. Pasini advocates its administration suspended in *adeps lanæ anhydricus* or *vaselinol*, but this is a cumbrous and inconvenient method. There seems to be no doubt that the bulk of the injection influences the pain to a great extent. The most satisfactory way, however, of minimising the pain is to give it intravenously, as is now advised by Professor Ehrlich himself, and this has the further advantage of accelerating the process of absorption, whereby the great desideratum of cure in one dose is made more likely. When given in this way the bulk of the injection must be largely increased in order to avoid the risk of intravascular thrombosis; 200 c.c. is the amount advised. Schreiber alone has practised this method ever since the introduction of the drug, but it does seem as if relapses are less frequent when it is employed than after intramuscular injections.

Is it, then, possible to sum up in a few sentences the present position in regard to this new arrival in the world of therapeutics? We may assert without fear of contradiction that a very powerful anti-syphilitic agent has been found which is going to establish for itself an important place in our armamentarium. It is more doubtful whether it will ever usurp the position so long and honourably held by mercury; many syphilologists of distinction, among whom may be cited Professor Lesser, Professor Gaucher, and Mr. Ernest Lane are of opinion that it will not do so, although against them we can pit the long lists of successfully treated cases already published by equally distinguished physicians, chief among whom is Wechselmann. At any rate, it is not a remedy to be employed lightly or without strict examination of the case to be treated, and sure information of the rather complex technique, neglect of which might lead the practitioner into distinctly awkward predicaments.

